# Seasonal Variations in Estimated Whole Blood Viscosity Associated with HbA1c: Evidence from Retrospective Pathology Review for Diabetes Management

**DOI:** 10.3390/ijms27010368

**Published:** 2025-12-29

**Authors:** Jovita I. Mbah, Phillip T. Bwititi, Lin K. Ong, Prajwal Gyawali, Ezekiel U. Nwose

**Affiliations:** 1 School of Health & Medical Sciences, Centre for Health Research, University of Southern Queensland, Toowoomba, QLD 4350, Australia; jovita.mbah@unisq.edu.au (J.I.M.); lin.ong@unisq.edu.au (L.K.O.); prajwal.gyawali@unisq.edu.au (P.G.); 2 School of Dentistry & Medical Sciences, Charles Sturt University, Wagga Wagga, NSW 2650, Australia; pbwititi@csu.edu.au

**Keywords:** seasonal variation, diabetes, whole blood viscosity, serum total protein, haematocrit and glycated haemoglobin

## Abstract

Elevation in the level of whole blood viscosity (WBV) is a known contributor to cardiovascular risk. Moreover, cardiovascular diseases are associated with seasonal variation and the potential impact of seasonal changes on blood viscosity, and associated biomolecules pose substantial cardiovascular risk and are therefore a subject of interest. To evaluate the effect of seasonal changes on whole blood viscosity, glycated haemoglobin and associated biomolecules, namely haematocrit and serum total protein, and their implications on management of diabetic cardiovascular risk are explored. This was a clinical laboratory retrospective observational study involving 10-year pathology data (1999–2008) which estimated whole blood viscosity (eWBV) and the associated biomolecules, namely haematocrit and serum total protein. Comparisons were made between seasonal changes and glycated haemoglobin, whole blood viscosity, haematocrit, and serum total protein levels. Whole blood viscosity, haematocrit, and serum total protein levels increased with colder seasons (*p* < 0.001), peaking in the winter. However, the seasonal variation in the level of glycated haemoglobin did not achieve statistical significance. Blood viscosity fluctuates between seasons, with peaks occurring in the winter season. This fluctuation will assist in adjusting monitoring and treatment strategies of diabetic risks seasonally. In addition, recognition of seasonal variations will help in precise risk assessment of timely interventions to mitigate the risk of cardiovascular events in diabetes management.

## 1. Introduction

Whole blood viscosity (WBV) is the resistance of blood flow through vessels and is influenced by factors such as haematocrit, plasma proteins, red cell properties of deformation, and aggregation [1,2]. Blood flow is closely related to viscosity, and a change in blood viscosity produces a threefold-greater inverse change in blood flow [3]. Alteration of blood flow can also instigate endothelial injury and promote vascular alteration through direct mechanical injury [4]. Hyperglycaemia initiates endothelial dysfunction and hypercoagulability and therefore impedes vascular function; exercise, on the other hand, was shown to raise coagulation potential not only in patients with cardiovascular disease (CVD) but also in healthy individuals, and these changes are associated with increased fibrinolytic activity [5].

Raised WBV has been linked with diabetes mellitus, hypertension, peripheral artery disease, and coronary artery disease, and it has been suggested that it might act in collaboration with atherosclerosis and microvascular malfunction in promoting an increase in vascular resistance related to a decreased capillary perfusion [6]. Understanding WBV is crucial because studies have shown that elevated WBV levels are linked to increased cardiovascular risk [7]. Some authors suggested that a more complex relationship exists between WBV and CVD for reasons such as small changes in blood viscosity above normal levels. Such changes have been linked to a decrease in blood pressure, an observation that has been explained as a consequence of stimulation of endothelium-dependent vasodilation, mediated by increase in shear stress [6].

Studies also highlighted that estimated whole blood viscosity (eWBV), which is derived from haematocrit and total protein levels, is associated with carotid thickening, thus suggesting that elevated WBV contributes to subclinical phase of atherosclerosis [8]; WBV is also an independent predictor of stroke and carotid atherosclerosis [9]. WBV plays a vital a role in the pathogenesis of various CVD, yet measuring WBV is widely underutilised. Analysing WBV might support a better understanding of the pathogenesis of different CVD such as hypertension and metabolic syndrome, which are not widely understood [10]. In clinical settings, eWBV is a practical approach to the assessment of blood viscosity due to availability and accessibility.

A phenomenon of interest is the relationship between CVD and seasonal changes. The association between environmental temperature and health has been known for a long time; for instance, there are reports suggesting higher CVD incidence in winter [11]. Some disorders are directly related to temperature extremes, and low seasonal temperatures increases the probability of death [12]. Seasonal peaks in respiratory, cardiovascular, and cerebrovascular mortality during winter is often referred to as “excess winter mortality” [13]. Further, patients admitted during winter are reported to have a longer length of stay in hospital and increased risk of all-cause mortality [14]. In Europe, seasonal mortality accounts for 16% more death in winter compared to summer [15].

Although multiple risk factors like physical inactivity, temperature, air pollution, and food habits possibly contribute to the increased risk of CVD during cold months [11], another factor could be an increase in WBV. An elevation in the level of WBV could be a result of higher levels of fibrinogen and low-density lipoprotein (LDL) cholesterol levels as well as high levels of vasoconstriction in the cold [16]. Studies have shown plasma viscosity, fibrinogen level, and factor VII activity to be raised during the winter period [17]. Reduced physical activity during winter, reduced blood flow, and decreased fibrinolytic activity are thought to be linked to the incidence of cardiovascular incidence during winter [11]. These result in a hypercoagulable state, which may increase cardiovascular morbidity and mortality [17,18].

Such seasonal changes in blood viscosity and red blood cell concentration are due to the effect of changes in temperature [19]. WBV is sensitive to changes in temperature, and a decrease in temperature from 36.5 °C to 22 °C has been associated with a 26.13% increase in WBV and 20.72% decrease in blood flow rate [20]. Cold spells were shown to have a short-term immediate effect and a longer lagged effect on plasma viscosity [21]. The importance of physical activity cannot be overemphasised as it has been shown to be a vital strategy for preventing CVD and all-cause mortality by improving blood glucose, lipid profile, insulin resistance, and vascular and endothelial function [22]. Air pollutants are other factors suggested to increase the incidence of CVDs. Air pollution is defined as a mixture of harmful substances, both particles and gases, released into the atmosphere because of human activities. Inhaling polluted particles is injurious to health, and the risk varies depending on the particle. The association of air pollution and respiratory diseases such as chronic obstructive pulmonary disease is well-known, but the relationship between air pollution and CVD is uncertain. However, a recent study suggests that the biggest health risk of air pollution is CVD [23]. Another study has shown that air pollution is lethal to the cardiovascular system, and high exposure to nitrous oxide is related to increased cardiovascular events and cardiovascular mortality the day after exposure [24].

Haematocrit (HCT) is a major determinant of blood viscosity [25]. An exponential relationship exists between WBV and HCT, particularly at higher levels of HCT, and the effect of HCT on WBV is higher in the veins, demonstrating low shear rates compared to the arteries’ high shear rate [26]. WBV is believed to be raised in diabetes, and this could be related to increased osmolarity, which causes increased permeability, thus increasing HCT and viscosity [27]. It has been reported that significant deviation in HCT from normal possibly constitutes cardiovascular risk even in healthy individuals [28]. HCT shows seasonal variability [29]; like WBV, a higher HCT has been reported in colder months compared to warmer months [29].

There is a paucity of literature on seasonal serum protein level variations in human. However, it was suggested that serum protein is maintained at a constant level, and seasonal changes are mainly due to haemodilution in summer and haemoconcentration in winter occasioned by changes in water content in the blood [30]. Seasonal higher C-reactive protein levels were reported in winter among healthy Koreans, and this is related to increased cardiovascular risk [31]. In addition to temperature and sunlight, other factors that impact seasonal variations include choice of food and physical activities [32].

Glycated haemoglobin (HbA1c) is a molecule formed by the attachment of glucose to haemoglobin. It has been established that as the average life span of red cells is 120 days, glucose continues to bind with haemoglobin depending on the level of glucose in the blood. Hence, HbA1c is being used as an indicator of glycaemia over the preceding 2–3 months that complement blood glucose level in diabetes diagnosis and management [33]. There has been ongoing research on diabetes management, but seasonal variations in HbA1c have yet to be considered. There have been reports that glycaemic control in diabetics as indicated by HbA1c levels shows seasonal patterns [34,35], with high values observed in cooler months [36,37]. However, in a recent study, seasonal variations with peak levels of HbA1c in warmer months were observed in Indian patients [38]. The potential impact of seasonal changes on HbA1c and WBV in diabetes management has not been extensively studied, and this knowledge is essential since environmental variations need to be considered in interpreting HbA1c results and formulating treatment strategy for diabetics.

***The gap in knowledge:*** The literature shows that several studies have reported seasonal variability in cardiovascular events with peaks in winter, and, indeed, a meta-analysis confirmed venous thromboembolism with a statistically significant peak in winter [11]. However, seasonal variations in the clinically assessed biomolecules (serum proteins) and HCT (surrogate test for another biomolecule) that determines changes in WBV and whether such variabilities corroborate related diabetes biomolecule (HbA1c) have yet to be exhaustively investigated. That is, the interplay between seasonal changes in HbA1c and eWBV in diabetes patients has not been thoroughly investigated.

***Statement of Objective:*** This study aims to evaluate seasonal patterns in HbA1c, eWBV, haematocrit, and serum total protein levels and explore their implications for diabetes management and cardiovascular risk.

## 2. Results

Demographics: The data comprised ‘N = 21,026’, including 11,165 males, 9854 females, and 7 who did not identify their gender. Data completeness was varied among the variables and particularly low for blood glucose tests (Table 1).

All four variables were lower during daylight savings compared to the standard time, and the differences are statistically significant except for HbA1c (Table 2). Comparison of the biomarkers of interest across the four seasons of spring, summer, autumn, and winter show a corroborating statistically significant difference in variables, except in HbA1c (Table 3).

The mean of the variables decreased from spring to summer (warm season) but increased from summer through autumn to winter (cold season). Data are normally distributed as indicated by skewness < 0.6 as previously reported [39]. The difference in variables across the seasons were statistically different *p* < 0.001, except for HbA1c *p* < 0.20 (Table 3).

HbA1c shows no difference across the four seasons, with *p* > 0.05, while eWBV levels were different across the same periods, with *p* < 0.05 (Table 4). Serum total protein levels were different in the seasons, showing *p* < 0.05, except for spring/autumn, showing *p* > 0.05; however, HCT showed a difference in other seasons, *p*< 0.05, except for spring/summer and autumn/winter, showing *p* > 0.05 (Table 5).

## 3. Discussion

This study examined seasonal variations in eWBV, haematocrit, serum total protein levels, and HbA1c among patients undergoing diabetes monitoring. The findings indicated that eWBV, haematocrit, and serum total protein levels significantly increase during colder seasons, peaking in winter. However, HbA1c levels showed similar seasonal fluctuation but did not achieve statistical significance. This seasonal elevation in eWBV, HCT, and serum total protein levels is consistent with previous studies reporting increased plasma viscosity and hypercoagulability in colder months [5,17]. The raised viscosity impairs blood flow and increases vascular resistance. The parallel increase in haematocrit and serum total protein supports this interpretation, given their role in determining eWBV.

The changes in levels of eWBV, HCT, and serum total protein from the daylight savings (warm) period and the standard time (cold) period were statistically significant at *p* < 0.000, except for HbA1c, showing *p* < 0.50; Table 2. This pattern was also observed when variables were compared between the four seasons; the variables decreased from spring to summer (warm season) but increased from summer through autumn to winter (cold season). Differences were statistically significant at *p* < 0.001, except for HbA1c at *p* < 0.20; Table 3. In a multivariate comparison, HbA1c showed no significance across the four seasons at *p* > 0.05. eWBV showed significant difference in all compared seasons at *p* < 0.05 while HCT was significantly different between the seasons at *p* < 0.001, except for spring/summer and autumn/winter at *p* > 0.05. In the same vein, total protein levels were different between seasons at *p* < 0.05, except for spring and autumn, showing *p* > 0.05 (Table 4 and Table 5).

The differences in eWBV across the four seasons with the peak in winter is linked to cardiovascular risk since increased viscosity slows blood flow, exerting more pressure on the heart to pump blood in the vascular system. Previous studies have shown plasma viscosity and proteins levels to be elevated in winter, creating a hypercoagulable state that leads to an increase in cardiovascular morbidity and mortality [17]. In addition to the impact of HCT and serum protein levels in determining WBV, red cell deformability, which contributes to blood viscosity, also showed a seasonal peak in winter and nadir in summer [17]. The combination of increase in HCT and serum protein levels, and potential haemoconcentration, leads to raised WBV, thus increasing the risk of CVD. Winter elevation of HCT is consistent with prior studies that reported raised levels after controlling for confounding factors [40], and the raised HCT was reported to be linked to hypertension and other cardiovascular risk factors [29]. A study in Congo reported elevated HCT during the cool seasons, and such observations could not be attributed to daily activities or diet, which changed minimally [29].

Summer reduction in HCT was thought to be due to haemodilution from increased environmental temperature and increased fluid intake, while haemoconcentration decreased plasma volume as well as physiological adaptation to increase the blood’s oxygen-carrying capacity, resulting tin elevated HCT in winter [41]. The lack of significant difference in HCT between spring/summer and autumn/winter could be due to marginal temperature differences between these seasons. At this juncture, it is pertinent to mention that there are opinions on considering temperature in the algorithm for eWBV [42,43]. Therefore, it has been cautioned that appropriate care must be taken in the interpretation of eWBV, especially considering the unit and factors utilised in the algorithm [44]. The data evaluated in this study is limited to de-identification information from the laboratory information system and without recourse to the individual. It would be interesting to investigate the expected seasonal effects in temperate vs. tropical regions in future studies.

There is a paucity of literature on seasonal serum protein level variations in humans; however, it was suggested that serum protein level is maintained at a constant level, and seasonal changes are mainly due to haemodilution in summer and haemoconcentration in winter occasioned by changes in water content in the circulating serum. This observation of a lack of significant differences in HbA1c could be explained from the point of view of HbA1c being an index of glycaemia in the preceding 2–3 months [33], meaning that samples tested in April (standard time) were estimating glucose concentration of February/March, which are in the warm season. Similarly, the values in spring reflect glucose concentrations in September/October, which are in the cold period, and the high values in winter reflect the glucose levels in June/July, which were in the cold period in the study. HbA1c level increasing from warm to cold seasons has previously been reported, and in countries where temperature variation is marginal, the variation in HbA1c is also minimal [36]. Other factors that could have affected fluctuations of HbA1c in diabetes include the age of circulating red cells [45] and possibly the effect of therapy [46]. The noted seasonal variation should be taken into cognizance in the interpretation of results in monitoring glucose levels in cold season.

Total protein levels were maintained at a constant level throughout the year, although haemodilution may lead to lower levels in summer [30]. Dehydration resulting from reduced water intake in winter and changes in water distribution within the body can concentrate serum proteins, leading to high levels. Serum total protein levels showed differences between seasons except for spring and autumn, which are transitional seasons. Serum protein levels’ fluctuations with seasons are reported to be mainly due to changes in water content and metabolic activities [30]. The lack of statistical significance in serum total protein in spring and autumn warrants further study.

### 3.1. Novelty of Findings

This study demonstrates that eWBV, haematocrit, and serum total protein levels show significant seasonal variations, with peak values occurring during winter. Variations in HbA1c did not achieve statistical significance, and this observation can be explained from the overlapping of seasons and the long-term approach to HbA1c measurement. These findings are clinically relevant because elevated blood viscosity in colder months may contribute to increased cardiovascular risk, particularly in individuals with diabetes [20].

There has been a dearth of literature regarding seasonal variation in total serum protein molecules; hence, this report is quite a contribution to evidence-based knowledge. The rise in haematocrit and serum total protein during winter suggests haemoconcentration and reduced plasma volume, likely due to lower fluid intake and physiological adaptation to increase the blood’s oxygen-carrying capacity [41]. These observed seasonal trends underscore the need to consider environmental context when interpreting blood viscosity and related biomolecules and managing cardiovascular risk.

### 3.2. Implications for Clinical Diagnosis and Research

There is evidence of high incidence of cardiovascular morbidity and mortality among the elderly in winter [17]. Increased blood viscosity during winter can have significant implications for cardiovascular health by increasing the risk of blood clots and associated complications, such as embolisms. Reduced plasma volume, as well as physiological adaptation to increase the blood’s oxygen carrying capacity, will lead to elevated HCT in winter [41]. The raised levels of the variables investigated in the current study during cold seasons invariably lead to increased blood viscosity. This highlights the importance of considering seasonal variations in the establishment of normal values for the variables and in the management and prevention of cardiovascular risk in diabetes.

It is worth mentioning that seasonal variations in the investigated biomolecules were not limited to diabetic patients. Studies of seasonal variation in haematocrit among healthy individuals, with the expectation of lower levels in summer compared to winter, have been reported [29,47]. It is also worthy to acknowledge and draw comparison to Doppler ultrasound that non-invasively assesses blood clot/flow. While eWBV is at no further cost, where routine HCT and serum protein tests have been performed, Doppler ultrasounds are relative expensive and/or unavailable, hence the issue of affordability.

In terms of molecular sciences research, many studies are ongoing but with less recourse to clinically established and conventionally available pathology tests. It has been known that HCT and serum proteins are factors to consider in molecular sciences of blood viscosity and its modulatory potential on gene transcriptions [48]. While researchers are becoming aware and adopting the eWBV method [49], it is imperative for molecular scientists to elucidate on clinically accessible pathology tests to bring research to practice. The significance of this lies in universal affordance of a clinical pathology service, not only in diabetes management but in other diseases that are complicated with blood flow pathophysiology.

In clinical practice, the relevance of eWBV evaluation is double-faceted. One aspect is that low levels would indicate risk of bleeding and contraindicate antiplatelet prophylaxis [50]. The second is that elevated eWBV despite stable HbA1c indicates risk of thrombosis, particularly regarding cardiovascular risk stratification in winter. A previous study’s dataset has indicated up to a 2.5% risk of bleeding or thrombosis [51], and that eWBV could be used for monitoring antiplatelet mediation as an international normalised ratio to monitor anticoagulant therapy [52,53]. What this report adds to the discourse is the evidence that eWBV is significantly higher in winter relative to summer (Table 1).

Further, from the perspective of currency and the relevance of this discourse, we have recently published related work on the correlation of eWBV with clinical parameters of a coagulation profile [54], which adds to the insight on gender differences in blood coagulability and viscosity [55,56]. There is also the exposition on WBV in the pathophysiology of blood flow and therapeutic potential [57], which expounds the discourse about cardiogenic dementia [58].

### 3.3. Strength and Limitation

The major strengths of this study are the large sample size and longitudinal design, spanning a decade of pathology data. This enhances the reliability and generalizability of the findings.

Limitations include the retrospective nature of the data and lack of information on comorbidities, weight or obesity status, medications, and lifestyle factors. However, eWBV is extrapolated from haematocrit and serum protein levels, which may not capture all determinants of viscosity such as red cell aggregation, deformability, and dehydration. It is pertinent to point out that other factors are subject to further analysis in future studies.

The potential for age, gender, or weight to influence seasonal variations in the pathology of molecules under focus has not been considered, hence acknowledged as limitations in the scope of this study. Indeed, it is acknowledged that the trio of age, gender, and obesity confound diabetes mellitus pathophysiology. While the association between obesity and seasonal variation is known to be complex, e.g., with links to levels of hydration and physical activities [22,40], age and gender are also speculated to confound seasonality in health [59,60]. The implication is that a regression analysis or other forms of predictive modelling would be required to account for possible confounding factors.

It is further acknowledged that eWBV does not provide a complete hemorheological assessment. Such a complete assessment may be impracticable as haemorrheology has typical complex factors of endocrine, humoral, and neural regulatory mechanisms [61,62]. For instance, humidity and temperature are speculated to influence blood viscosity [43], although these could also be part of seasonal variations.

In addition, there are genetic factors, such as those seen in thalassaemia, that can disrupt iron homeostasis [63], which could impact haemostasis. Religious fasting is yet another factor that impacts changes in meal timing, hydration status, sleep patterns, and physical activity amongst others, all of which influence glycaemic control and cardiovascular risks [64]. Yet, it is pertinent to note that this study is contributing to what has been ongoing for 21 years [65] and is still continuing [39,66]. That is, the evaluation of all confounding factors of haemorrheology is outside the scope of this work, but regression analysis with routine clinical data is necessary to support individualised care concepts in interpretations of laboratory results.

## 4. Materials and Methods

***Design:*** This was a clinical laboratory-based observational study using a retrospective longitudinal archived dataset of clinical pathology [50,67,68]. This study assessed seasonal variation in eWBV and associated biomolecules, haematocrit and serum total protein levels, among patients undergoing diabetes monitoring.

***Setting:*** Albury area of South-West regional New Wales (NSW) of Australia. The public pathology service covers South-Western regional NSW and environs.

***Ethics considerations:*** Ethical approval is not required as secondary data were collected and used for this study. Primary data were neither collected nor used in this study.

***Participants:*** De-identified data of patients who attended the pathology service for laboratory tests of the parameters of interest, i.e., HbA1c and determinants of eWBV, namely haematocrit and serum total protein levels. Of the 21,026 patients that participated in this study, 11,165 were male, 9854 females, while 7 were of unidentified gender.

***Selection criteria:*** Inclusion criteria were patients that attended the laboratory more than once and had at least two results at different dates for data variables of interest spanning across the four seasons between the period of 1999–2008. Conversely, the exclusion criteria were patients with less than two results of data variables of interest, as this does not allow comparison.

***Variables:*** This includes clinically assessed biomolecules (HbA1c and serum protein), with HCT being a surrogate test of haemoglobin molecule level, and the dependent variable eWBV (from HCT, serum protein level). The discretional choice of HCT was based on the fact that eWBV is derived from it. Others are seasons, winter, summer, autumn, spring, as well as daylight savings and standard time as independent grouping variables.

***Data source/measurement:*** Public pathology service in Albury, 10 years (1999–2008) of archived clinical pathology data generated from laboratory information systems [68]. For each patient’s record, the following data were extracted: demography (age and sex), laboratory parameter HbA1c, haematocrit and serum total proteins, and date of test (used to assign data to season).

***Study size:*** The total number of participants in this study is n = 21,026 of HbA1c data, which have results for haematocrit and serum protein levels.

***Groupings:*** Two seasonal groupings of daylight savings and standard time, as well as four seasonal subgroupings of spring, summer, autumn, and winter; hence, a total of six groups.

1. Daylight savings: 1st Sunday of October—1st Sunday of April (1 October–31 March)
2. Standard-time: 1st Sunday of April–1st Sunday of October (1 April–30 September)
3. Spring: October–December (1 October–31 December)
4. Summer: January–March (1 January–31 March)
5. Autumn: April–June (1 April–30 June)
6. Winter: July–September (1 July–30 September)

***Statistical analysis:*** These included univariate and analysis of variance (MANOVA) tests. Univariate analysis compared between daylight savings vs. standard time and between the four seasons. Multivariate analysis, MANOVA, was used to compare biomolecules (HCT + serum total proteins) and eWBV with the four seasons. A two-tailed *p* value < 0.05 was considered statistically significant. The statistical tool used is IBM SPSS statistics version 29. It should be acknowledged that this study was strictly focused on seasonal variations in de-identified pathology data. Therefore, grouping is based on defined seasons, and there is, albeit discretionally, no requirement for cluster analysis.

## 5. Conclusions

There is eWBV fluctuation between seasons with the peak values in winter. This elevation in winter is applicable to other biomolecules of HCT, total protein and HbA1c. However, the difference in HbA1c did not achieve statistical significance, perhaps due to overlapping in seasons due to HbA1c’s long-term view of glycaemia. Considerations of seasonal fluctuations in the establishment of reference values and observations assist to adjust monitoring and management strategies of diabetic risks seasonally. In addition, recognition of seasonal variations will help in result interpretation and precise risk assessment of timely interventions to mitigate the risk of cardiovascular events in diabetic patients. Knowledge of these seasonal variations and related environmental factors are vital in devising efficient and robust public health strategies for controlling the risks of cardiovascular events. As decreased physical activities during cold seasons reduce blood flow, alternative ways of increasing physical activities during the colder seasons should be sought. Further research should explore seasonal variations in blood viscosity using direct measurements and must include broader clinical variables such as medication use, physical activity, and hydration status. Prospective studies can validate these findings and assess the impact of seasonal interventions on cardiovascular outcomes in diabetic populations.

## Figures and Tables

**Table 1:** Descriptive statistics of variable biomolecules.

Variables Continuous	N	Min	Max	Mean	Std Dev
HbA1c levels	21,016	2.40	21.90	6.85	1.77
Serum protein levels g/L	21,011	34	138	71.56	5.74
Haematocrit (decimal fraction)	21,016	0.13	0.66	0.42	0.04
Estimated whole blood viscosity	21,011	8.99	25.82	16.89	1.23
Random blood glucose level	5452	0.60	86.30	9.61	6.99
Fasting blood glucose level	12,384	0.40	34.20	7.49	3.20

**Table 2:** Univariate analysis for daylight savings vs. standard times of the variables.

Variable	Group *	Mean	Std. Deviation	N	*p* Value
HbA1c	1	6.8439	1.73342	10,041	0.500
	2	6.8604	1.79619	10,974	
Serum Protein	1	71.26	5.789	10,039	0.000
	2	71.83	5.677	10,971	
Haematocrit	1	0.42128	0.044685	10,041	0.000
	2	0.42529	0.044880	10,974	
eWBV	1	16.81838	1.226140	10,039	0.000
	2	16.96229	1.225274	10,971	

* 1: Daylight savings AEDT; 2: Standard time AEST.

**Table 3:** Univariate analysis of the variables with four seasons.

Variables	Seasons (Groups)	Mean	Std. Deviation	N
HbA1c (*p* < 0.20)	3 Spring	6.870	1.753	4668
	4 Summer	6.821	1.716	5373
	5 Autumn	6.840	1.762	5163
	6 Winter	6.878	1.826	5812
Serum Protein (*p* < 0.001)	3 Spring	71.425	5.957	4667
	4 Summer	71.123	5.637	5372
	5 Autumn	71.469	5.596	5162
	6 Winter	72.149	5.730	5810
Haematocrit (*p* < 0.001)	3 Spring	0.422	0.045	4668
	4 Summer	0.421	0.044	5373
	5 Autumn	0.425	0.045	5163
	6 Winter	0.425	0.045	5812
eWBV (*p* < 0.001)	3 Spring	16.854	1.266	4667
	4 Summer	16.787	1.189	5372
	5 Autumn	16.903	1.215	5162
	6 Winter	17.015	1.232	5810

**Table 4:** Multivariate comparison of variables with four seasons—HbA1c and eWBV.

Dependent Variable	(I) **	(J) **	I − J *	Sig.	95% Confidence Interval	
					Lower Bound	Upper Bound
HbA1c levels	3	4	0.0491	0.165	−0.0202	0.1184
		5	0.0299	0.402	−0.04	0.0998
		6	−0.0088	0.799	−0.0769	0.0592
	4	3	−0.0491	0.165	−0.1184	0.0202
		5	−0.0192	0.577	−0.0867	0.0483
		6	−0.0579	0.083	−0.1235	0.0076
	5	3	−0.0299	0.402	−0.0998	0.04
		4	0.0192	0.577	−0.0483	0.0867
		6	−0.0387	0.252	−0.105	0.0275
	6	3	0.0088	0.799	−0.0592	0.0769
		4	0.0579	0.083	−0.0076	0.1235
		5	0.0387	0.252	−0.0275	0.105
Estimated whole blood viscosity	3	4	0.06666	0.007	0.01862	0.11471
		5	−0.04867	0.049	−0.09717	−0.00018
		6	−0.16114	<0.001	−0.20833	−0.11395
	4	3	−0.06666	0.007	−0.11471	−0.01862
		5	−0.11534	<0.001	−0.16213	−0.06854
		6	−0.22781	<0.001	−0.27325	−0.18236
	5	3	0.04867	0.049	0.00018	0.09717
		4	0.11534	<0.001	0.06854	0.16213
		6	−0.11247	<0.001	−0.15839	−0.06655
	6	3	0.16114	<0.001	0.11395	0.20833
		4	0.22781	<0.001	0.18236	0.27325
		5	0.11247	<0.001	0.06655	0.15839

* Positive values mean [I] > [J]; while negative values mean [I] < [J]. ** 3—spring, 4—summer, 5—autumn, 6—winter.

**Table 5:** Multivariate comparison of variables with four seasons—HCT and serum protein.

Dependent Variable	(I) **	(J) **	I − J *	Sig.	95% Confidence Interval	
					Lower Bound	Upper Bound
Serum protein levels g/L	3	4	0.30	0.008	0.08	0.53
		5	−0.04	0.716	−0.27	0.18
		6	−0.72	<0.001	−0.94	−0.5
	4	3	−0.30	0.008	−0.53	−0.08
		5	−0.34	0.002	−0.56	−0.13
		6	−1.03	<0.001	−1.24	−0.81
	5	3	0.04	0.716	−0.18	0.27
		4	0.34	0.002	0.13	0.56
		6	−0.68	<0.001	−0.9	−0.47
	6	3	0.72	<0.001	0.5	0.94
		4	1.03	<0.001	0.81	1.24
		5	0.68	<0.001	0.47	0.9
Haematocrit level in decimal fraction	3	4	0.00128	0.153	−0.00048	0.00304
		5	−0.00347	<0.001	−0.00524	−0.00169
		6	−0.00317	<0.001	−0.0049	−0.00145
	4	3	−0.00128	0.153	−0.00304	0.00048
		5	−0.00474	<0.001	−0.00646	−0.00303
		6	−0.00445	<0.001	−0.00611	−0.00279
	5	3	0.00347	<0.001	0.00169	0.00524
		4	0.00474	<0.001	0.00303	0.00646
		6	0.00029	0.733	−0.00139	0.00197
	6	3	0.00317	<0.001	0.00145	0.0049
		4	0.00445	<0.001	0.00279	0.00611
		5	−0.00029	0.733	−0.00197	0.00139

* Positive values mean [I] > [J]; while negative values mean [I] < [J]. ** 3—spring, 4—summer, 5—autumn, 6—winter.

## Data Availability

The original contributions presented in this study are included in the article. Further inquiries can be directed to the corresponding author.
